# Application of evidence on probiotics, prebiotics and synbiotics by food industry: a descriptive study

**DOI:** 10.1186/1756-0500-7-754

**Published:** 2014-10-23

**Authors:** Mary N Mugambi, Taryn Young, Reneé Blaauw

**Affiliations:** Division of Human Nutrition, Faculty of Medicine and Health Sciences, Stellenbosch University, Stellenbosch, South Africa; Centre for Evidence-Based Health Care, Faculty of Medicine and Health Sciences, Stellenbosch University, Stellenbosch, South Africa

**Keywords:** Food industry, Infant formula, Evidence, Probiotics, Prebiotics, Synbiotics

## Abstract

**Background:**

This study assessed how the food industry applies the knowledge and evidence gained from synbiotics, probiotics or prebiotics research in infants, on the general paediatric population. This study also explored: what happens after the clinical trials using infant formula are completed, data is published or remains unpublished; the effectiveness and type of medium the formula manufacturers use to educate consumers on probiotic, prebiotic or synbiotic infant formula.

**Findings:**

This was a descriptive study (a survey) that used a structured questionnaire. All listed companies that manufacture and / or market food products with added probiotics, prebiotics or synbiotics for infants were identified and invited to participate. People responsible for research and development were invited to participate in the survey. A letter of invitation was sent to selected participants and if they expressed willingness to take part in the study, a questionnaire with a written consent form was sent. Descriptive statistics and associations between categorical variables were to be tested using a Chi-square test, a p < 0.05 was statistically significant.

A total of 25 major infant formulas, baby food manufacturers were identified, invited to participate in the survey. No company was willing to participate in the survey for different reasons: failure to take any action 5 (20%), decision to participate indefinitely delayed 2 (8%), sensitivity of requested information 3 (12%), company does not conduct clinical trials 1 (4%), company declined without further information 4 (16%), erroneous contact information 6 (24%), refusal by receptionists to forward telephone calls to appropriate staff 3 (12%), language barrier 3 (12%), company no longer agrees to market research 1 (4%).

**Conclusion:**

Due to a poor response rate in this study, no conclusion could be drawn on how the food industry applies evidence gained through probiotics, prebiotics or synbiotics research on infants for the benefit of the general paediatric population. More information and greater transparency is needed from the infant formula manufacturers on how they apply the evidence gained from the research on probiotics, prebiotics and synbiotics on infants.

## Background

Scientific evidence from numerous studies in the last 25 years confirms that breastfeeding is the optimal way to feed infants, since breast milk contains all the essential nutrients to meet babies’ needs, as well as antibodies that fight off infection [1–4]. The World Health Organisation (WHO) estimates, that if women breastfed their infants, up to 1.5 million infant deaths or 13% of deaths in children under 5 years old could be prevented annually [5]. Despite the benefits of breastfeeding, more women are choosing formula feeding, either exclusively or giving mixed feeds (both formula and partial breastfeeding). Globally, this has resulted in sales of infant formula skyrocketing creating stiff competition among infant formula companies to manufacture new and innovative products [5].

A factor driving research and innovation in the infant food industry is the need to understand the composition and functional characteristics of breast milk. Therefore, scientists continuously conduct research to identify how infant formula can be adapted to more closely resemble the composition and function of human milk. This has resulted in different components being added to infant formula such as docosahexaenoic acid (DHA), arachidonic acid, synbiotics, probiotics or prebiotics [6–9].

### Probiotics, prebiotics and synbiotics

Probiotics are defined as “live microorganisms” which when administered in adequate amounts may confer a health benefit to the host. [9–11] The main probiotics used worldwide are Lactobacillus and Bifidobacteria which are found in the gastrointestinal (GI) microflora [11, 12]. Formula companies have been adding probiotics to infant formula [11–16].

Prebiotics are non- digestible food ingredients that may benefit the host by selectively stimulating the growth and/or activity of one or a limited number of bacteria in the colon and improving the host’s health [9, 10, 17, 18]. The most widely studied prebiotics are inulin and fructooligosaccharide (FOS) which are added to different foods as fat and sugar replacements to improve texture or for their functional benefits [9, 10]. The latter is why formula companies now add prebiotics to infant formula. Adding prebiotics to formula stimulates the growth of only beneficial bacteria in the gastrointestinal tract to levels found in breastfed infants [17–19].

When probiotics and prebiotics are administered simultaneously, the combination is termed Synbiotics [9, 10, 19]. A new trend in the infant food industry is the addition of synbiotics to infant formula.

### How strong is the evidence for adding probiotics, prebiotics and synbiotics to infant formula?

There is evidence that a healthy GI microflora in infants is necessary to achieve optimal health and growth [20]. For infants who are not breastfed, there is a rational to adapt infant formulas to promote an intestinal microbiota resembling that of breastfed infants, which has greater concentration of bifidobacteria, fewer potentially pathogenic bacteria than formula fed infants. This is achieved by adding probiotics, prebiotics or synbiotics to infant formula for full term and preterm infants [11]. Adding these ingredients to infant formula changes the intestinal microbiota of infants [19, 21, 22].

Systematic reviews of randomized clinical trials offer the highest level of evidence for information on the effectiveness of an intervention [23–25]. Systematic reviews on full term infants given probiotics show certain strains of probiotics improve stool consistency and frequency (*Lactobacillus GG*) [26], other strains increase average formula intake (*L. reuteri, B. lactis*) [22], and support normal growth (*B. lactis, B. longum* BL999, *L. rhamnosus LPR, Lactobacillus* GG, *L. reuteri* ATCC 55730) [26]. For preterm infants, administration of probiotics results in reduced risk of Necrotising Enterocolitis (from combinations of *Lactobacillus bifidus, streptococcus thermophillus,* and *bifidobactrium infantis*) and mortality (*L acidophilus* and *B infantis*) [27].

Prebiotics have a good safety record at levels found in existing food components. Flatulence or abdominal bloating are reported at doses greater than 20 g/day. Abdominal cramps or diarrhoea are reported at doses greater than 50 g/day [19]. Adding prebiotics to formula stimulates the growth of only beneficial bacteria in the gastrointestinal tract to levels found in breastfed infants and improves intestinal architecture which reduces intestinal permeability [12, 19, 28–31].

### Communication of best evidence to the consumer

The environment for communicating health and nutrition information has changed in recent years due to an increase of television, radio channels, internet usage, and new media such as social networking sites, podcasts and webinars [32]. To communicate with the consumers, the food industry uses multiple channels to promote and sell their products with a goal of achieving profitable growth. The food industry uses subtle messages of better nutrition as part of their promotional activities [33]. In the context of probiotics, prebiotics containing food products, the consumer may not understand the meaning or importance of scientific terms such as probiotics, *Lactobacillus*, fructooligosaccharide or inulin. Thus, there is a great need for clear information in a language that the consumer can understand.

### Rationale for research

To our knowledge, there are no studies that have assessed how the food industry applies the knowledge and evidence gained from research on probiotics, prebiotics or synbiotics on the general paediatric population. This study attempted to explore what happens after research trials using infant formula have been conducted and the data is published or remains unpublished. Based on the new scientific evidence, do the companies routinely develop and market a new probiotic, prebiotic or synbiotic containing infant formula, or improve on one that is already sold on the market?

Probiotic infant formulas have been sold in Europe and Asia in the last 15 years but are not used widely in North America [34]. A physical check of several retail outlets in the Western Cape, South Africa, yielded few brands (sometimes only two) of probiotic containing infant formula. Yet several companies (in collaboration with academic institutions) have conducted research projects using probiotics and prebiotics on infants in Southern Africa [35, 36]. There is little or no information on the differences between the study formula and the retailed product. It is not clear how the manufacturers of probiotic, prebiotic or synbiotic containing infant formula educate the consumers on their products. This study set out to answer product specific questions on genera of probiotics used, product viability at end of shelf life, differences between study and retailed product. As well as explore the effectiveness and type of medium the infant formula manufacturers use to educate the consumers on probiotic, prebiotic or synbiotic infant formula.

Safety issues are also an area of concern. The two probiotic infant formula brands available in the Western Cape, South Africa retail outlets state that using water with temperatures above 40°C (degrees centigrade) will compromise the natural cultures. This contradicts the WHO “Guidelines for safe preparation, storage and handling of powdered infant formula” which recommends that water with a minimum temperature of 70°C should be used to minimize the risk of potentially deadly infections caused by *Enterobacter Sakazakii*, bacteria that has been found in infant formula [37]. In addition there is a lack of published evidence on clinical benefits from long term use of probiotic containing infant formula [26, 38]. This study tried to explore how the infant formula companies address the contradiction to WHO guidelines on formula preparation and safety issues of long term usage of probiotic infant formula.

### Research question

Does the food industry apply the evidence gained through probiotics, prebiotics and synbiotics research on infants for the benefit of the general paediatric population?

### Research aim

To investigate how the infant food industry applies evidence gained through probiotics, prebiotics and synbiotics research on infants.

### Objectives

The objectives of this study were to determine the following:

*Application of evidence*:

1. If new research evidence resulted in new infant formula products been developed,
2. If there were any differences in study and retailed infant formula,
3. The frequency of conducting research using probiotics, prebiotics or synbiotics containing infant formula.

*Publication of results:*

4. If the infant formula companies had intentionally NOT published study results that were viewed as negative or having no clinical benefit to infants?
5. If study results perceived to be negative, were withheld and was new research conducted to confirm the results?

*Medium for consumer education:*

6. The type and effectiveness of medium used to educate the consumer,
7. The presence of bias in promoting formula feeding more than breastfeeding.

*Compliance to WHO guidelines:*

8. How formula companies complied with WHO guidelines on formula preparation with a focus on high water temperature and its effects on probiotics, synbiotics containing infant formula?

*Safety of long term use of probiotic or synbiotic containing infant formula,*

9. How companies addressed safety, since there is a lack of published evidence on the clinical benefits of long term consumption of probiotic containing formula (longer than 1 year).

*Product viability,*

10. If the probiotic, synbiotic containing infant formula remain viable throughout storage or are there substantial changes in the number of colony forming units at the end of shelf life?

*How companies keep abreast of the latest research on probiotics, prebiotics and synbiotics in infant formula and weaning foods?*

11. If the formula companies had staff designated to keep track of research or was it on “ad hoc” basis?

## Methodology

### Study design

This was a descriptive study (a survey) employing the use of a structured questionnaire developed by the researcher.

### Company selection

Companies that manufacture and/or market food products with added probiotics, prebiotics or both (synbiotics) for infants and children were identified through several databases such as EBSCOhost, Business Source Premier and DATAMONITOR^360^. In addition, company websites were visited to acquire the contact information of individual companies. The person/people responsible for research and development were invited to participate in the survey. Study participants included clinical research managers and individual researchers in the infant food companies. Worldwide, the numbers of infant food companies (especially infant formula manufactures) are few. Therefore all listed companies were invited to participate in the study. The number of study participants per company was one or two.

### Data collection and processing

A letter of invitation was sent to selected participants, inviting them to take part in the study. The letter of invitation explained all aspects of the study, and if they expressed willingness to take part in the study, a questionnaire with a written consent form was sent via post, email or fax. If the questionnaire was posted, a stamped envelope was included for returning the completed questionnaire to the researcher. A maximum of four reminders were given to the participants to complete the questionnaire. The participants were free to withdraw from the study at any time without any consequence.

Due to the expected small sample size, maintaining anonymity of study participants with the corresponding company name was difficult. Therefore, data processing was done according to product and company name. However, during report writing, all identifying details (name of study participant, product and company name) were excluded. Only the researcher and statistician had access to the data.

### Questionnaire description

A questionnaire was designed for this study based on relevant published information. The questionnaire focused on product specific questions, research based questions, education of consumers and safety issues. It was validated for content by sending it to experts in the field of probiotics, prebiotics and infant nutrition, who were able to judge if the questionnaire met the objectives of the study. These experts did not partake in the study nor were they associated with the infant food industry.

### Data analysis

Researchers planned to enter the collected data into SPSS (Statistical Program for Social Sciences) for analysis. The data was to be analysed using descriptive statistics and associations between categorical variables, be tested using a Chi-square test. A p < 0.05 was considered statistically significant. A statistician was consulted at every step of the study process.

### Ethics approval

Ethics approval to conduct this study was given by the Human Research Ethics Committee at the University of Stellenbosch, Faculty of Medicine and Health Sciences, reference number N11/07/203.

## Findings

A total of 25 major infant formula and baby food manufacturers were identified from around the world and invited to participate in the survey (Table 1). A total of 5 (20%) companies initially agreed to participate but took no action by not signing the informed consent form and completing the questionnaire. The decision to participate in the study was delayed indefinitely for 2 (8%) companies since their head of department was too busy to make a final decision. Sensitivity that the requested information would give the competition an advantage was cited by 3 (12%) companies for not participating in the study, while 1 (4%) company stated they manufacture baby food and distribute it for retail without conducting any clinical trials. A total of 4 (16%) companies declined to participate without giving any further information. Erroneous contact information given on company websites hindered any contacted being made with 6 (24%) companies. Company representatives from 3 (12%) companies refused to forward telephone calls from the researchers to the appropriate department and staff. Three (12%) companies cited language barrier (Mandarin, German, Dutch) as a reason for not participating in the study, despite offers to professionally translate the study documents into a language of their choice. One (4%) company stated that it was overwhelmed with people making requests for market research, as a result it had restructured and “market research was no-longer a priority” (Table 2). In the end no company was willing to participate in the survey.

**Table 1:** **List of 25 baby food companies and infant formula manufacturers invited to participate in survey**

Company name	Company name
Abbott Laboratories/Abbot Nutrition	Milupa
Aspen Phamarcare	Morinaga Milk industry Co. Ltd
Beech-nut nutrition corporation	Nestle (South Africa and Switzerland)
Danone baby and medical nutrition BV	Organix brands
Earth's Best (Hain Celestian Group)	Pfizer Inc (SA) and Pfizer Head office
FrieslandCampina (Netherlands)	Raptakos Brett & Co. Ltd.
Gerber products company	SMA Nutrition (Ireland and UK)
Hangzhou Beingmate Group Co Ltd.	Synutra International
HiPP GmbH & Co Vertrieb KG	Tiger brands
JH J Heinz	Wakodo Co. Ltd
Kewpie	Wockhardt Limited
Mead Johnson	Hero AG
Meiji Dairies

**Table 2:** **Reasons for not participating in survey**

Reason(s) for not participating in survey	N =25
	Number of companies n (%)
No Action taken by company after agreeing to participate in survey	5 (20%)
Head of department too busy to make decision	2 (8%)
Requested information too sensitive - may give competition an advantage	3 (12%)
Company does not conduct clinical trials, just manufacture infant food, distribute it for retail	1 (4%)
No reason given for declining to participate in survey	4 (16%)
Researchers unable to make contact with company through use of internet (emails, “contact us” features in company websites), telephone, fax or post office.	6 (24%)
Company receptionist/contact person refused to forward call/put researchers in touch with appropriate person to answer questions	3 (12%)
Quote: “Too many people conducting market research on company, company has other priorities than answering market research questions.”	1 (4%)
Language barrier – “prefer questionnaire in local dialect” such as Mandarin, Dutch, German.	3 (12%)

Note: Several companies gave more than one reason for not participating in survey.

## Discussion

To our knowledge, this was the first study to explore how the food industry applies evidence gained through research on probiotics, prebiotics and synbiotics on infants for the benefit of the general population. As a direct result of the poor response rate in this survey, several study objectives and key questions remain unanswered. These are discussed below.

### Application of evidence

Despite more than 30 years of research on probiotics, prebiotics or synbiotics on infants and children, any differences between studied and retailed infant formula such as the strains of probiotic bacteria used could not be established. It remains unknown if new evidence from clinical trials led to the improvement of existing formula, development of new infant formula or weaning foods containing probiotics or synbiotics.

### Publication of results (Publication bias)

Publication bias is defined as “the tendency for investigators, journal editors and reviewers to submit or accept a manuscript for publication based on the directions or strength of the study findings [39]. Publication bias can have far reaching consequences on the public. For example, if an intervention that is not effective is falsely considered effective and administered to patients, an effective treatment that is available is withheld. Not publishing results from research where the intervention is discovered to be harmful; may indirectly harm study participants taking part in future research. This is because other investigators will (unknowingly) repeat the same research, testing the harmful intervention, causing suffering on a different group of people [39]. This study was not able to establish if companies engaged in research had intentionally NOT published study results that were viewed as negative or not having any clinical benefits to infants and children. Whether companies conducted new research to confirm results that may have been perceived as negative could not be established.

### Medium for consumer education

The type and effectiveness of medium used to educate the consumer on probiotics, prebiotics or synbiotics containing formula or baby foods could not be established. The numerous techniques used by the formula and baby food industry to increase awareness of their products are beyond the scope of this study and are described elsewhere. Only one education and promotion technique is illustrated below.

#### Internet

The internet is an important source of health information for parents [40–42]. Company websites offer advice on infant feeding, child rearing and health care issues. Some websites have useful product information, most websites use information on breastfeeding to jump to the second best option; formula feeding [3].

Most websites of formula manufacturers have product specific content concerning infant formula brands. Websites present images of branded packs linked with information about specific infant formula. These website links are accessible to the public, health and medical professionals. Research has shown consumers (mothers) get confused with formula advertising [40]. In situations where infant formula and follow-on formula share brand identities, consumers recall advertising and messages for follow-on formula and think it also applies to infant formula. As a result, information and promotional messages designed around follow-on formula are transferred to infant formula products. This type of confusion has far reaching implications [40].

Navigating the websites of the 25 companies invited to participate in this study, in addition to the product specific content in the websites, only eight companies had brief descriptions of probiotics or prebiotics, five companies had health claims on probiotics and one company had a health claim on prebiotics. There was no mention of the strains of probiotics or type of prebiotics in their products. In addition, the information on probiotics and prebiotics was difficult to obtain from the websites and could be inaccessible to consumers without advanced computer skills, tertiary education or sufficient knowledge on what to look for.

### Compliance to WHO guidelines

The position of formula companies on how they comply with WHO guidelines on water temperature during formula preparation could not be established. WHO recommends diluting the powdered formula in water at a temperature of at least 70°C to inactivate *cronobacter spp.* (*Enterobacter sakazakii*) [37]. South Africa’s “Regulations Relating to Foodstuffs for Infants and Young Children, Government Gazette number 35941” state that labels for any infant formula, follow-up formula must “provide instructions for appropriate use according to the latest FAO/WHO guidelines.” The gazette requires the labels to state that infant formula is not always sterile and may contain harmful microorganisms, emphasizing appropriate preparation [43]. Yet the labels of infant formula found in retail stores of Western Cape, South Africa do not recommend using water above 40°C.

The European Society of Pediatric Gastroenterology, Hepatology and Nutrition (ESPGHAN) committee on nutrition and French Food Safety Agency (AFSSA) disagree with WHO guidelines and state that heating water to temperatures greater than 70°C is not necessary and maybe harmful to the nutritional quality of formula. Using hot water (greater than 70°C) may lead to formation of curds, risk of severe burns and the loss of 10 to 25% of some nutrients: Thiamine, Vitamins B1, B6, B12, Folic acid, and Vitamin C [44, 45]. The effect of water temperature on *Cronobacter spp.* (*Enterobacter sakazakii*) is striking. At 37 to 39°C, there is optimal growth, at 5.5 to 8°C there is minimal growth. At room temperature, *Cronobacter spp.* has the potential for rapid growth [44, 45]. It is worth noting the rate of contamination with *Cronobacter spp.* has decreased over the years from 14% in 1980s to 2.4% in mid 2000s [44–46].

### Safety of long term use of probiotics or synbiotics containing formula

The way companies address the question on safety of long term consumption of probiotics, synbiotics of infant formula could not be established. Safety of long term use is an important issue since majority of consumers of probiotics, synbiotics containing formula and baby foods use these products for more than a year. According to ESPGHAN committee on nutrition, there is a lack of published evidence on the clinical benefits and safety from long term consumption of probiotic containing formula [26, 38]. How the formula and baby food companies educate the consumer on this issue is yet to be determined or observed.

### Product viability

Whether bacteria in retailed probiotics or synbiotics containing infant formula remain viable throughout shelf life was not established in this study. There are few reports on the stability of probiotics in powdered formula for infants and toddlers [47]. Several studies have conducted long term stability tests on bifidobacteria in powdered formula and results show the viability of live bacteria (such as bifidobacteria) decreased with length of time in storage and with increase in temperature [47, 48]. Consumers usually store powdered formula at room temperature. However, the formula may be exposed to high temperatures during transportation, during hot seasons or, in countries with hot weather conditions. If there is a large reduction in viable cell counts of probiotic bacteria, the commercial use of the formula is lost and the consumer does not benefit from the expected probiotic effects [47]. The change in stability at various storage temperatures should be made clear by formula manufacturers.

### How companies keep abreast of the latest research on probiotics, prebiotics and synbiotics in infant formula and weaning foods

How companies keep abreast of the latest research on probiotics, prebiotics and synbiotics in infants could not be established. This study tried to find out if there are any formal mechanisms in place to ensure that employees or researchers keep abreast of the latest research. The formula and baby food industry needs to be more transparent on this issue.

### Limitations

#### Sampling frame

Only online electronic databases were used to identify the companies around the world that manufacture infant food products with probiotics, prebiotics or synbiotics. Small regional companies that were not listed in the electronic databases were missed and subsequently not invited to participate in the study. Different methods could have been used to identify small regional companies.

#### Selection bias (under-coverage bias)

Efforts were concentrated on inviting people responsible for research and development such as clinical research managers and individual researchers. Other staff such as product managers could have been invited to participate in the study.

#### Survey participation rates, non-response bias

Survey participation rates were nil. Many company staffs were cautious after the initial contact and invitation to participate in the study. After continued dialogue, they were unwilling to participate in the survey. During telephone conversations with the some company employees, the researchers were perceived to be in collaboration with the competition.

## Conclusion

Due to a poor response rate, no conclusion could be drawn on how the food industry applies evidence gained through probiotics, prebiotics or synbiotics research on infants and children for the benefit of the general paediatric population. More information with greater transparency is needed from the infant formula and baby food companies on how they apply the evidence gained from the extensive research conducted using probiotics, prebiotics and synbiotics on infants and children.

## Abbreviations

AFSSA: French Food Safety Agency
°C: Degrees centigrade
DHA: Docosahexaenoic acid
ESPGHAN: European Society of Pediatric Gastroenterology, Hepatology and Nutrition
FOS: Fructooligosaccharide
GOS: Galactooligosaccharide
SPSS: Statistical Program for Social Sciences
WHO: World Health Organisation.
